# Panofskyan iconological reading of images representing nursing in Brazilian philately*

**DOI:** 10.1590/1980-220X-REEUSP-2024-0284en

**Published:** 2025-02-07

**Authors:** Anesilda Alves de Almeida Ribeiro, Genival Fernandes de Freitas

**Affiliations:** 1 Universidade de São Paulo, Escola de Enfermagem, Departamento de Orientação Profissional, São Paulo, SP, Brazil.

**Keywords:** Nursing, history of nursing, science in the arts, drawing, philately, Enfermería, historia de la enfermería, ciencia en las artes, dibujo, filatelia

## Abstract

**Objective:**

To analyze the images of Brazilian postal documents depicting nursing.

**Method:**

Historical research with a time frame between 1843 and 2022. The data was collected using the visual observation technique, from the iconographic collection of the Post Office Museum and philatelic catalogs. The postal drawings were analyzed using Erwin Panofsky's Iconological Method.

**Results:**

62 postal documents alluding to nursing were identified. The graphic art was made up of elements of the profession's symbolism, personalities, social organizations, professional categories, care actions, health institutions, nursing professional associations, scientific events and nursing schools. There was an absence of academic themes, pioneering nurses and nursing journals.

**Conclusion:**

Since 1934, nursing has been represented in Brazilian philately, regardless of the political context. The representations show public recognition by the Brazilian state of the social importance of the profession and of nursing professionals in human health care. The study innovated nursing research in Brazil by using the postage stamp as a source of data and the Panofskyan methodology for reading images of the profession.

## RESUMEN

**Objetivo:**

Analizar las imágenes de documentos postales brasileños que representan la enfermería.

**Método:**

Investigación histórica con un marco temporal entre 1843 y 2022. Los datos se recogieron mediante la técnica de observación visual, a partir de la colección iconográfica del Museu dos Correios y de catálogos filatélicos. Los diseños postales se analizaron mediante el Método Iconológico de Erwin Panofsky.

**Resultados:**

Se identificaron 62 documentos postales alusivos a la enfermería. El arte gráfico estaba compuesto por elementos de la simbología de la profesión, personalidades, organizaciones sociales, categorías profesionales, acciones asistenciales, instituciones sanitarias, asociaciones profesionales, eventos científicos y escuelas de enfermería. Hubo una ausencia de temas académicos, enfermeras pioneras y revistas de enfermería.

**Conclusión:**

Desde 1934, la enfermería está representada en la filatelia brasileña, independientemente del contexto político. Las representaciones muestran el reconocimiento público del Estado brasileño de la importancia social de la profesión y de los profesionales de enfermería en el cuidado de la salud humana. El estudio innova la investigación sobre enfermería en Brasil al utilizar el sello postal como fuente de datos y la metodología panofskyana para la lectura de imágenes de la profesión.

## INTRODUCTION

The image is a representation and a vehicle of communication, having function, history and meaning. Within the history of art, different topics can be represented by images and constructed using various techniques. The theme of nursing has been explored by countless visual artists, with the image of the profession being expressed in a realistic and stylized way in figurative works of art, such as painting, sculpture, photography, engraving, watercolor, illustration and drawings, especially those printed on postage stamp.

The postage stamp was created in 1840 in England to serve as proof of payment when sending correspondence. Brazil was the second country in the world to issue a postage stamp, on August 1, 1843. The Brazilian postage stamp is printed at the Brazilian Mint and issued by the Brazilian Enterprise of Posts and Telegraphs (ECT), which is linked to the Ministry of Communications. ECT issues other postal documents, such as commemorative postmarks, post cards, first day covers (FDC) and commemorative sheets. It is up to the National Philatelic Commission, made up of representatives of the federal government and the public institutions mentioned above, to choose the themes that will be encompassed in the yearly Postage Stamp Program. The images are created by artists and are made up of textual and graphic elements and filled in with colors ranging from monochrome to multicolored^(1)^.

Until the middle of the 20th century, postage stamp served exclusively as collectors’ items and philatelic commercialization products. Since 1997, they have been used as a source of research data. An integrative literature review identified the use of postage stamps by Brazilian researchers in various sciences, but no studies in the area of health. The national production, consisting of articles, course completion papers, monographs, dissertations and theses, highlights the value of the postage stamp in academic research, as it contains the record and disseminates ideas and relevant social, scientific and historical events^(2)^.

Universal postal history has recorded the image of various professions, including nursing. Nursing philatelic designs have been used by international researchers since 1941. The first scientific article on this subject was published in the *American Journal of Nursing*, in the United States of America (USA), and featured a Brazilian postage stamp^(3)^. A study shows that the first representation of a real and nominally identified nurse in Latin American philately was in 1957^(4)^. A biographical article on an American laic nurse was illustrated with a postage stamp featuring her portrait^(5)^. Religious nurses, military nurses and nurses who founded nursing schools were honored on postage stamps^(6)^. A study in Costa Rica identified the history of local nursing on postage stamps^(7)^. Venezuelan philately disseminated the nursing identity of religious, humanitarian and hospital institutions^(8)^. Studies of Argentinian philately show different ways of representing nursing^(9)^, and a minority of female nurses and physicians compared to male physician^(10)^.

From Europe, the philatelic studies of nurse and philatelist Dr. Maria Teresa Miralles Sangro stand out, published in the form of an article, book and doctoral thesis. By analyzing her collection of postal documents, she identified historical facts, personalities, commemorative dates, uniforms, symbols and nursing care on postage stamps from more than 130 countries, including Brazil^(11,12,13,14)^.

For more than 80 years, the Brazilian postage stamp has served as an object of scientific study in international medical and nursing research. Despite this, there has still been no study of Brazilian nursing on the image of the profession on ECT postage stamps. Against this background, a study was carried out with the guiding question: what are the representations of nursing in Brazilian philately and what is their meaning? Objective: to analyze the images of Brazilian postal documents depicting nursing^(15)^.

## METHOD

### Type of Study

Research with a historical approach, in the field of History of Health Sciences and the domain of History of Nursing.

### Location, Population, Selection Criteria and Sample Definition

The research was carried out in Itajubá, MG, Brazil and on the internet. The population comprised all postal documents issued by ECT. The selection criteria included Brazilian postal documents issued between 1843 and 2022, with images featuring elements alluding to the nursing profession. When defining the sample, postal documents with a repeated image or slight variation in the design or caption, those related to disease and those without a representation of nursing were excluded.

### Data Collection

The data was collected by the first author, from January to December 2022, using the Visual Observation Technique, in her private collection of postage stamps and printed material from the Zioni-Soares Catalog^(16)^ and the Brazilian Stamp Catalog^(1)^, and in the digital collection of the Post Office Museum.

### Data Analysis and Processing

The analysis was guided by the theoretical-methodological framework of the German art historian Erwin Panofsky (1892–1968), created for the study of meaning in the Visual Arts, which allude to everyday reality. Panofsky says that a work of art is a representation that reflects its creator’s worldview and the historical context in which it was created. Panofsky’s framework uses two concepts: Iconography, relating to the description and classification of the constituent elements of the artistic composition; and Iconology, which deals with the interpretation of the intrinsic message, i.e. the total meaning of the work of art^(17)^.

Data processing followed the Iconological Method created by Erwin Panofsky^(17)^, characterized by the sequential application of three categories: 1) Pre-Iconographical Description, 2) Iconographical Analysis; and 3) Iconological Interpretation. The study began with a description of the constituent elements of the image and their relationships; followed by an analysis of the theme of the representation, identifying the creator and the historical context of graphic art, the biography of personalities, the history of the organization or institution represented; and ended with an interpretation of the meaning of the visual representations of nursing in Brazilian philately.

### Ethical Aspects

The research did not involve human beings. The project was cleared by the Research Ethics Committee of the School of Nursing of the University of São Paulo, Opinion No. 4.420.440, in line with Resolution No. 510/2016 of the National Health Council on the use of images. The Brazilian Post Office’s Philately Management authorized the use and reproduction of the images for research and publication purposes^(15)^.

## RESULTS

A total of 17,670 postal documents were methodically observed (11,144 postmarks, 4,930 postage stamps, 1,302 FDCs, 249 post cards and 45 sheets). The application of the inclusion criteria selected 156 images and the exclusion criteria resulted in a sample of 62 postal documents: 31 commemorative postmarks, 22 postage stamps, five FDCs, two commemorative sheets and two post cards^(15)^.

The images chosen for the study were classified by similarity, giving rise to four thematic groups: 1) nursing personalities and social organizations (15 images), 2) nursing courses and schools (eight images), 3) scientific events and nursing professional associations (26 images), and 4) nursing in care actions in health institutions (13 images).

Due to space limitations, the most symbolic images from each thematic group are presented, grouped into four figures, distributed and analyzed according to the chronological order in which they were issued by the ECT. The other images in the sample and other information can be found in the thesis document^(15)^.

### Nursing Personalities and Social Organizations

**Figure 1:**
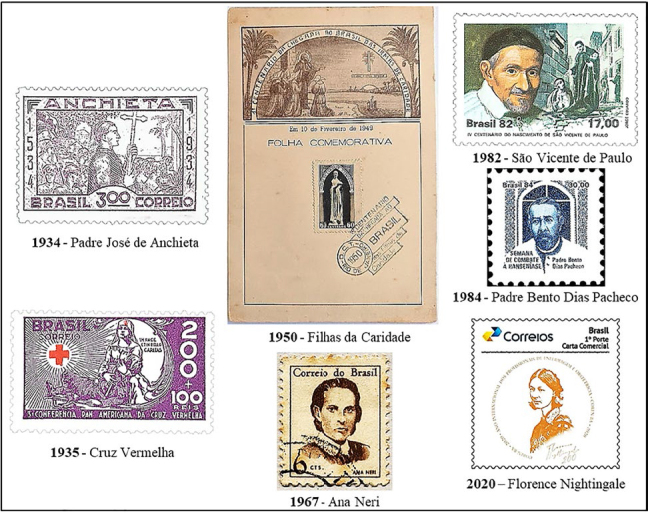
Postal images of nursing personalities and social organizations.

Source: Brazilian Post Office.

Brazilian philately represented five nursing personalities, three Brazilian (Priest José de Anchieta, Ana Justina Ferreira Neri and Priest Bento Dias Pacheco) and two foreign (St. Vincent de Paul and Florence Nightingale), and two nursing social organizations: the Red Cross and the Daughters of Charity (Figure 1).

Priest José de Anchieta was born in 1534 in Spain, studied in Portugal with the Society of Jesus, came to Brazil in 1553, where he worked as a catechizer, teacher, writer and Nurse, and died in 1597. Anchieta is recognized by nursing history literature as Brazil’s first Nurse^(18)^. The 4th centenary of his birth, celebrated in 1934, included the issue of a postage stamp. The image, created by Leopoldo Alves Campos, depicts this Catholic saint wearing a cassock, carrying a processional cross and providing spiritual care to indigenous people on the Brazilian coast. ECT has issued others Anchieta postage stamps, all praising his role as a priest. An Argentine study also found that historical figures in the health sector are not always portrayed in philately because of their profession, but because of other activities, including religious ones^(10)^.

The Red Cross (Cruz Vermelha) social organization was created in 1863 in Switzerland and is now present in 190 countries, with the Brazilian unit being founded in 1908. The 3rd Pan-American Red Cross Conference, held in 1935 in Rio de Janeiro/RJ, was the subject of a postage stamp. The image, created by Marino Ferreira Pinheiro, features a war scene, with a stylized Nurse, wearing a long uniform with an apron and veil, supporting a wounded soldier in her arms. The graphic art is considered beautiful, unusual and a symbol of the ideals of nursing, of welcoming and caring for others, and is recurrent in international scientific literature, especially in that which presents postage stamps alluding to nursing^(3,11,12)^.

The Daughters of Charity (Filhas da Caridade), a Catholic woman’s religious organization created in 1633 in France by St. Vincent de Paul and Luiza de Marillac, spread to five continents, arriving in Brazil on February 10, 1849. The 1st Centenary of the arrival of these nuns in the country was celebrated with a commemorative sheet issued in 1950. The image, created by Bernardino da Silva Lancetta, depicts the Daughters of Charity wearing long robes and horned veils. In the context of the postal issue, Brazilian Vincentian nurses were dedicated to health education, home care for the poor and sick, headed health institutions and ran Catholic confessional nursing schools^(18)^.

Ana Neri was born in Bahia in 1814 and died in Rio de Janeiro in 1880. She provided nursing services to the Brazilian Army’s health corps during the Paraguayan War (1864-1870), a conflict in which some of her brothers and sons took part^(18)^. This link with the Armed Forces earned her a tribute in 1967, when ECT issued a postage stamp and an EPD with the caption: Ana Justina Ferreira Neri - 1st Nurse of Brazil. The postage stamp image, created by Waldomiro Puntar, features a portrait of this illustrious Brazilian nursing personality. The context of the issue was the Military Dictatorship (1964–1985) and the tribute to Brazil’s Famous Women. Despite being issued on May 12, the press release does not mention the celebration of Nurses’ Day, but presents the biography of this celebrity, the only Brazilian Nurse to have been identified nominally and visually on a postage stamp to date.

The 4th Centenary of the birth of St. Vincent de Paul (1581–1660), celebrated in 1982, also featured a postage stamp. The image, created by Jorge Eduardo Alves de Souza, is made up of a reproduction of a 17th century painting by Simon François, showing the figure of this Catholic priest caring for a sick and homeless French citizen. The press release provides a biographical summary of this saint of the Catholic Church, a member of the Order of St. Francis of Assisi, who dedicated his life to charity, created male and female congregations such as the Lazarist Priests and the Daughters of Charity, and spread his Vincentian charism of charitable care for the poor in 150 countries, including Brazil^(18)^.

The 1984 Week to Combat Leprosy featured the issue of a postage stamp with the effigy of Priest Bento Dias Pacheco (1819–1911). The image, created by Martha Poppe, consists of the profile of this Catholic priest wearing a cassock and the stained glass window of a church. The nursing literature is unaware of the work of this historical personality, but the Brazilian State, through the Brazilian Post Office, awarded him the title of Nurse, for the care provided to leprosy patients in Itu/SP, as described in the press release.

Florence Nightingale was born in 1820 and died in 1910. This English Nurse organized the British nursing service in the Crimean War (1853–1856), founded modern nursing and the world’s first nursing school based on scientific technical rigor. She disseminated the profession and nursing care through letters, notes, reports and books^(9,18)^. The bicentenary of her birth, celebrated worldwide in 2020, has been publicized in Brazil through the issue of a postage stamp. The image consists of his portrait and signature. The artwork was provided by the Regional Nursing Council of Bahia (Coren-BA). It is a personalized postage stamp, issued to order, with normal circulation, nationwide, in the autarchy’s correspondence and for sale to philatelists and collectors.

### Nursing Courses and Schools

**Figure 2:**
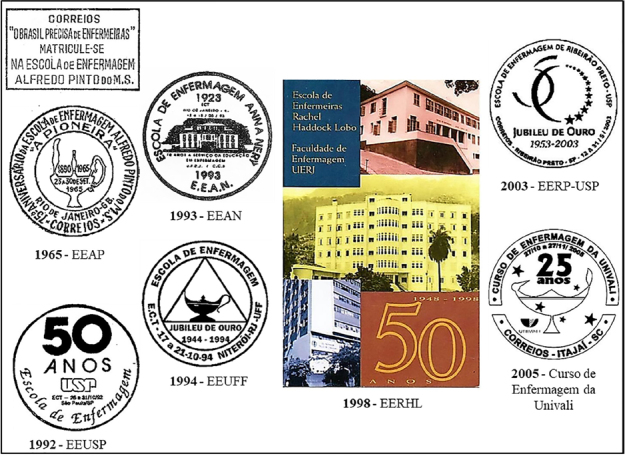
Postal images of nursing courses and schools.

Source: Brazilian Post Office.

ECT issued postal documents for seven public or private, federal or state Higher Education Institutions (HEIs) (Figure 2).

The Alfredo Pinto Nursing School (EEAP) was created in 1890 on the French model, with doctors directing and teaching. In 1943, this federal public HEI in Rio de Janeiro, a pioneer in the training of male and female nurses, hired nurses as directors and teaching staff^(18)^. In September 1965, ECT issued two EEAP commemorative postmarks. The rectangular postmark bears a caption stating the shortage of nurses in the country and the opening of enrollment at the school. The circular postmark was issued in celebration of the School’s 75th anniversary, and the image consists of the drawing of a lamp, alluding to the lamp that symbolizes nursing. These postal issues inaugurated the marketing of nursing schools through Brazilian philately.

The University of São Paulo School of Nursing (EEUSP), created in 1942, had as its first director Nurse Edith de Magalhães Fraenkel (1889-1968), a USA graduate^(19)^. Since its beginnings, this state HEI has been a center of excellence in professional training and research in nursing. EEUSP’s 50th anniversary, celebrated in 1992, saw the issue of a commemorative postmark, with an image made up of the celebratory logo, consisting of the USP Logo, without the presence of nursing symbols or the school’s visual identity - Logo and Coat of Arms. In the context of its Golden Jubilee, the institution celebrated 25 years of the Journal of School of Nursing – University of São Paulo (REEUSP) and inaugurated the Historical-Cultural Center for Iberian American Nursing (CHCEIA), responsible for preserving the School’s historical memory.

The Anna Nery Nursing School (EEAN) was created in 1923, with the aim of graduating female nurses and spreading the Anglo-American model of modern nursing education nationwide, which was characterized by having nurses in charge of the school and classrooms, and teaching in a boarding school. Until 1931, the institution was run by American nurses, after which Brazilian nurses took over. In 1937, the school was incorporated into the Federal University of Rio de Janeiro (UFRJ). In 1971, it began offering a mixed nursing course^(18)^. The 70th anniversary of the EEAN, celebrated in 1993, saw the issue of a commemorative postmark. The image features a reproduction of the facade of the Classes Pavilion, the scene of great struggles and achievements for Brazilian nursing.

The School of Nursing at the Fluminense Federal University (EEUFF), created in 1944, had as its first director Nurse Aurora de Afonso Costa (1903–1999), under whose management the federalization and implementation of specialization courses took place. In 1994, in celebration of its Golden Jubilee, this center was renamed the Aurora de Afonso Costa Nursing School (EEAAC) and ECT issued a commemorative postmark to mark its 50th anniversary. The image consists of the geometric figure of a triangle and the nursing lamp inside.

The Rachel Haddock Lobo School of Nurses (EERHL) was created in 1948, named honoring Rachel Haddock Lobo (1891–1933), a pioneering Brazilian Nurse who graduated in France and completed her studies at EEAN. In 1968, this HEI was renamed the Faculty of Nursing of the State University of Rio de Janeiro (UERJ). The commemoration of the school’s 50th anniversary in 1998 included the issue of a post card. The image, created by Carlota Rios, is made up of the facade of the school’s three headquarters. The caption on the back of the issue states that this HEI has contributed to health, development and citizenship in Rio de Janeiro since its creation.

The Nursing School of Ribeirão Preto, at USP (EERP-USP), created in 1953 as an annex to the Ribeirão Preto Medical School, had as its first director Nurse Glete de Alcântara (1910–1974), a Toronto, Canada graduate. By virtue of its bylaws, at times this institution was run by physicians and engineers. Since 1986, it has been run by nurses. The 50th anniversary of EERP-USP, celebrated in 2003, saw the issue of a commemorative postmark. The image, created by Solange Aparecida da Silva and Rogério Cândido Ribeiro, consists of the school’s fiftieth anniversary logo, made up of an arch with 13 stars of different sizes, without the nursing symbol or the beautiful institutional logo. In the context of the Golden Jubilee, the 10 years of the Latin American Journal of Nursing (RLAE), published by the School, were celebrated.

The 25th anniversary of the Nursing Course at the University of Vale do Itajaí (Univali), celebrated in 2005, was commemorated with the issue of a commemorative postmark. The image, created by Nelenize Heyse Moreira, consists of Univali’s logo, five stars and the light bulb on the model created to make up the logo of the Brazilian Nursing Association (ABEn), which was used improperly and in a fragmented way, because the image was created by order of the ABEn to serve as its trademark^(15)^. Univali is a private HEI, based in Itajaí/SC, made up of the School of Health Sciences, of which the Nursing Course was the pioneer. As part of the course’s Silver Jubilee, ECT also issued a personalized postage stamp which was applied to the diplomas of that year’s graduates.

### Scientific Events and Nursing Professional Associations

**Figure 3:**
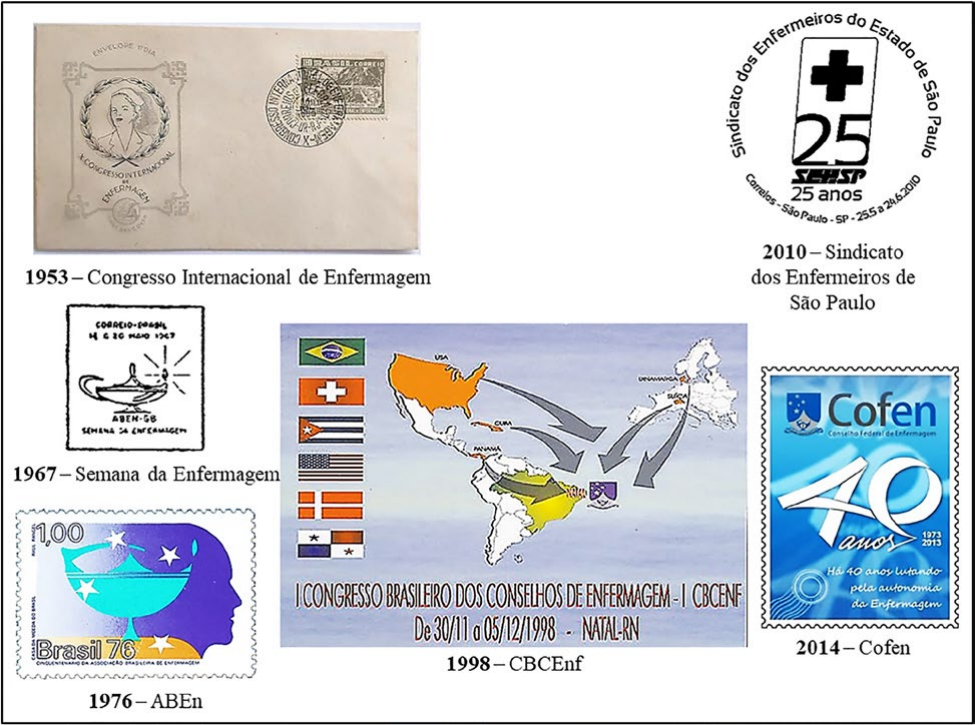
Postal images of scientific events and nursing professional associations.

Source: Brazilian Post Office.

The Brazilian postal service issued postal documents with images alluding to three scientific events and three Brazilian nursing professional associations (Figure 3).

The 10th. International Nursing Congress, promoted by the International Council of Nurses, held in 1953 in Rio de Janeiro, included an FDC encompassing a postage stamp and a commemorative postmark. The FDC image, created by Waldomiro Puntar, features an anonymous modern Nurse wearing a white uniform and a Nurse’s Cap, flanked by green leaves. The stamp, in green, features a drawing of the Rio Petrópolis highway, the Serra do Mar mountain range and the Greek lamp. The caption on the commemorative postmark gives the date of the event. These postal issues mark the first record in Brazilian postal history of two symbols of nursing – the light bulb and the color green - and two symbols socially related to the profession – the white uniform and the Nurse’s Cap.

The Nursing Week is the first scientific event in Brazilian nursing. It was instituted in 1940 by Laís Netto dos Reys, director of EEAN, and is celebrated from May 12 to 20^(18)^. The event is part of the commemorative calendar of educational and health institutions, and its program includes tributes to illustrious professionals, cultural and scientific activities and promotion of the profession^(18)^. The 1967 ABEn-Guanabara (ABEn-GB) Nursing Week featured a commemorative postmark, in square format, with an image of a Greek lamp with a lighted flame and rays.

The ABEn, created in 1926, is the first professional association for Brazilian nursing, responsible for the cultural, scientific and political promotion of the profession. Its first president was Edith de Magalhães Fraenkel^(18)^. The 50th anniversary of the ABEn, celebrated in 1976, included the issue of a postage stamp. The image, created by Raul Rangel, consists of the profile of the Nurse and the ABEn logo, made up of the Greek lamp with the flame burning and four stars, alluding to the Southern Cross Constellation.

The Brazilian Congress of Nursing Councils (CBCENF) is an event organized by the Federal Nursing Council (Cofen) and Regional Councils (Coren) or the Cofen/Corens System^(20)^. The first edition, held in 1998, featured a post card. The image is made up of a representation of the maps and flags of Brazil, Panama, Cuba, USA, Denmark and Switzerland, the participants’ countries of origin. Arrows indicate the city where it was held: Natal/RN. The promoting entity is indicated by the Cofen coat of arms, created by Ori Ramos, which consists of the lamp of nursing, the serpentine baton of health and five stars, alluding to the five regions of the country where the Corens units are located.

The 25th anniversary of the São Paulo State Nurses’ Union (Sindicato dos Enfermeiros de São Paulo, SEESP), celebrated in 2010, also saw the issue of a commemorative postmark. The image, created by RS Press Comunicações, is made up of the Greek cross, formed by two arms of the same size that cross in the center, at a right angle.

Cofen, created in 1973, is responsible for overseeing the professional practice of Brazilian nursing. Its first president was Nurse Maria Rosa Sousa Pinheiro (1908–2002), a Toronto, Canada graduate^(20)^. Cofen’s 40th anniversary, celebrated in 2014, included the issue of a postage stamp. The image, created by Sandy de Assis Andrade, consists of the celebration logo, made up of the numeral 40 drawn in a stylized way and the Cofen coat of arms. The image has been filled in with blue, although Cofen has established green as the symbol of the nursing profession.

### Nursing in Care Actions in Health Institutions

**Figure 4:**
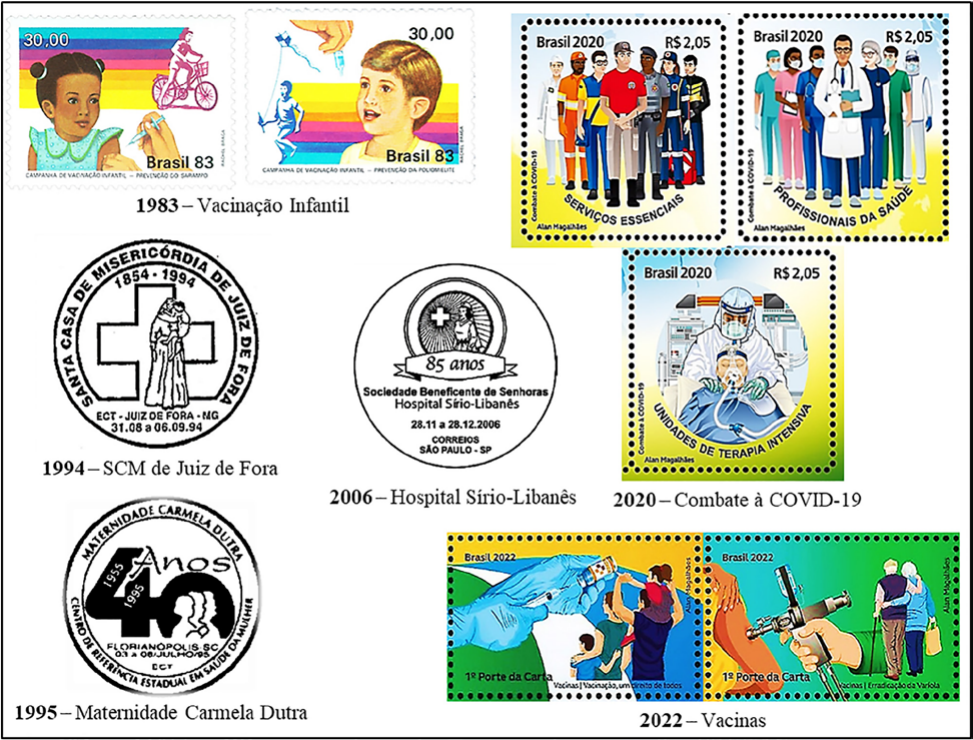
Postal images of nurses providing care in health institutions.

Source: Brazilian Post Office.

ECT issued postal documents on nursing care provided in Primary Health Care (PHC) and secondary and tertiary care provided in hospitals, private or public (Figure 4).

The images on the two child vaccination postage stamps, created in 1983 by Rachel Braga, are made up of a representation of the Nursing Technician’s hand. The identification of this professional category was based on the provisions of the Professional Practice Law, which states that the administration of medication is the responsibility of the Nursing Technician^(20)^. The images show her administering the measles vaccine to a girl, intramuscularly, using a glass syringe; and administering the oral poliomyelitis vaccine, using a drop of the immunizer dripped directly into a boy’s mouth. These postage stamps were used by the Ministry of Health, in partnership with the Ministry of Communications and ECT, to publicize vaccination campaigns, with the aim of raising awareness among families and increasing vaccination coverage among the population. By adhering to the National Immunization Program, PHC nurses would meet the target set in the Vaccination Calendar and the Ministry of Health would control vaccine-preventable diseases^(18)^.

The Holy House of Mercy of Juiz de Fora (Santa Casa de Misericórdia de Juiz de Fora), created in 1854, is linked to the Brotherhood of Our Lord of the Stations. In the beginning, the institution’s nursing service was run by Catholic religious nurses from the Congregation of St. Catherine, originally from Germany. Later, nursing management was handed over to laic nurses^(15)^. The institution celebrated its 140th anniversary in 1994 with a commemorative postmark. The image consists of the institution’s logo, made up of the Greek cross and a religious Nurse wearing a white habit, veil, crucifix and a child in her arms.

The Carmela Dutra Maternity Hospital (Maternidade Carmela Dutra) was founded in 1955 in Florianópolis/SC. This public health institution relied on the chaplaincy service of Jesuit priests, the management of the Sisters of Divine Providence and midwives in the delivery room. In the 1960s, the first qualified religious nurse was hired. In 1972, a laic obstetric nurse joined the nursing service^(21)^. The 40th anniversary of the maternity hospital, celebrated in 1995, saw the issue of a commemorative postmark. The image consists of the midwife or obstetric nurse, flanked by an obstetrician and a mother holding her child in her arms.

The Ladies Charitable Society of the Syrian-Lebanese Hospital (Hospital Sírio-Libanês) was set up in 1921 by Adma Jafet and ladies from the Arab community in São Paulo, with the aim of supporting the hospital, a private institution of excellence, staffed by qualified Nursing Technicians and Nurses who provide quality and safe nursing care to patients^(15)^. The Society celebrated its 85th anniversary in 2006 with a commemorative postmark. The image, created by the Ogilvy Brasil advertising agency, features a reproduction of the institution’s first logo, made up of the Greek cross, rays and a Nurse in a white long-sleeved uniform, apron and veil.

The images on the three COVID-19 postage stamps, created in 2020 by Alan Magalhães, are made up of representations of non-real-life health professionals, wearing colored pants and shirts and closed shoes. The postage stamp above and to the left features the image of a white first-aid Nurse wearing the blue uniform of the Mobile Emergency Care Service (SAMU), made up of overalls with the star of life and reflective stripes, a cap and a red backpack. The postage stamp on the right shows the Nursing Assistant, black, wearing a pink uniform, goggles, mask and clipboard; the Nursing Technician, white, wearing a dark green uniform and mask; and the Nurse, white, wearing a white uniform and Personal Protective Equipment (PPE): mask, Face Shield, goggles and gloves. The postage stamp below shows a white intensive care Nurse caring for a patient in the Intensive Care Unit, flanked by advanced life support equipment and materials.

The images on the two Vaccine postage stamps were created in 2022 by Alan Magalhães. The one on the left depicts the gloved hand of a Nursing Technician aspirating immunizer from a vaccine vial using a disposable syringe and a family of four. The image on the right is made up of the hand of the Nursing Technician in a green uniform, with a gun and a bottle of immunizer attached, administering the smallpox vaccine to the teenager’s right arm and flanked by an elderly couple. The presence of people of different ages alludes to everyone’s right to the vaccine.

## DISCUSSION

The results show that there were no postal issues on the subject of nursing during the government of Pedro II (1840–1889) and the First Republic (1889–1930). From the 1930s onwards, nursing was a recurring theme in Brazilian philately, regardless of the political context. The first image, dated 1934, depicts Priest José de Anchieta. In 1935, there was a representation of a fictional or imaginary Nurse, in a stylized image alluding to the Red Cross.

In 1967, a real Nurse was identified visually and by name on a postage stamp: Ana Neri. Only one modern Brazilian Nurse, i.e. a graduate, had her name recorded on a postal document: Rachel Haddock Lobo, whose nominal identity was related to an HEI. A study of postage stamps from the American continent identified women from various professions and occupations, including nurses, physicians, queens, heroines, writers, athletes, engineers and government officers^(4)^.

The term Nurse (male and female) appears on postage stamps press release in 1935 and 1985, alluding to the Red Cross and Priest Bento Dias Pacheco, respectively.

The elements used to compose the representations of nursing were: the Cofen coat of arms, the ABEn logo and the symbols of the profession: light bulb, syringe, snake, cross and the color green, which is a recurring professional symbol in philately worldwide^(8,12–14)^. The Nurse’s Cap and the color blue were secondary symbols alluding to nursing. The lamp, the professional uniform and the Nurse’s Cap were represented in variations of shape, model and color. Graphic elements unrelated to the profession were used, such as a bow, star and lightning bolt. The greatest diversity of compositional elements was seen in the images related to ABEn. The largest number of broadcasts were about the Cofen/Corens System, but with repetition of the institutional coat of arms. The most realistic images were those reproducing photographs of historical personalities and the facades of HEI headquarters.

Brazilian postal history has recorded laic female Nurses and war volunteers, as well as religious white male Nurses, who lived in a period when nursing was a practical activity and lacked scientific knowledge^(1,18)^. The representations of Nursing Personalities and Social Organizations show the role of nursing in the context of peace and war. The image of Florence Nightingale, St. Vincent de Paul, the Daughters of Charity and the Red Cross are recurrent in international philately, due to the recognition of their works and individual and collective contributions to the promotion of human health^(4–14)^.

Brazilian philately has issued postal documents celebrating the anniversary of Nursing Courses and Schools, sharing national pride in their longevity and their role in propagating science, innovation and technology in the profession. These representations reveal the importance of nursing HEIs in training new generations of care, management, teaching and research professionals. Worldwide philately has also paid tribute to pioneering and centenary nursing courses and schools^(12–14)^.

ECT paid tribute to the Brazilian Nursing Professional Associations and publicized their events by issuing postal documents telling the story of associativism, fiscalization, trade unionism and professional scientificity. These representations reveal the contribution of the nursing professional associations to the scientific, cultural, political, ethical, legal and trade union promotion of nursing. Worldwide philately has also issued postage stamps publicizing associations, international events and historical facts in nursing^(4–14,22)^.

The Brazilian Post Office highlighted the nursing work carried out by black and white professionals from different categories, in different contexts and scenarios. The representations highlight the leading role of nursing and the value of the care provided in the prevention, promotion, recovery and rehabilitation of health, at all stages of the life cycle and with the use of advanced technology. Care actions are also recurring themes in world philately, highlighting PHC, rural, specialized and hospital nursing^(3–14)^.

In the context of the 2020 and 2022 postal issues, Brazilian nursing was experiencing the vicissitudes of the COVID-19 pandemic, with a high morbidity and mortality rate among professionals^(23)^ and the rise of the anti-vaccine movement. Nursing was effective on the front line in confronting and fighting the disease, despite the abrupt changes and loss of life, and for this it received several tributes through postage stamps. Due to the unprecedented nature and seriousness of the viral disease, the Brazilian state used this media to pay tribute to the professionals on the front line of the pandemic and to spread the value of vaccination in preventing diseases, including against COVID-19, reinforcing the idea that vaccines save lives.

Despite being valued and part of the world’s philately^(3–14,22)^, Brazilian postal documents^(1,16)^ lacked some nursing-related themes. There was a lack of portraits and names of real professionals from the 20th and 21st centuries; commemoration of the centenary of the births of pioneers and nursing theorists; the ritual of the Oath of the Profession, the Reception of the Insignia and the Passing of the Lamp; the fiftieth anniversary of nursing journals and graduate programs; the presidents of ABEn and Cofen councilors; the Brazilian Nursing Congress (CBEn); celebration of the creation of the Professional Practice Law and the Nursing Code of Ethics, Nurses’ Day and Nursing Technician and Assistant’s Day; classrooms, events and academic games; care environments and ritualistic practices such as administering blood products, collecting material, dressings, bandaging, checking vital signs, physical examination; Red Cross bracelet; martyred and indigenous Nurses.

A limitation of the study is the fact that all the postal documents issued by the Brazilian postal service since 1843, as well as the press releases, have not been viewed, due to their lack of preservation by the ECT and the Post Office Museum. And the lack of information on the history, visual identity and celebratory logos of some educational and health institutions and nursing professional associations.

The research contributed to an understanding of the process of choosing the themes of postal documents and the importance of nursing being represented in this type of media, the profession being recognized by Brazilian philately as a theme of social relevance and historical interest. Priest Bento Dias Pacheco was presented as a novelty of professional identity, but his biography merits further investigation. The study brings as an innovation to Nursing History Research in Brazil the use of postal documents as data sources and the Panofskyan methodology in reading images of the profession.

## CONCLUSION

Brazilian postal history has celebrated the nursing profession in a special way, disseminating its main historical marks through realistic, symbolic and fictional images. Despite the late appearance and absences observed, the representations gave visibility to the professional identity, disseminated the scientificity, symbolism and history of Brazilian nursing, paid homage to and immortalized: 1) Nursing Personalities and Social Organizations, 2) Nursing Courses and Schools, 3) Scientific Events and Nursing Professional Association, 4) Nursing in Care Actions in Health Institutions. The artistic expressions about nursing, materialized in Brazilian postal documents, circulated the world and contributed to the creation of a collective visual memory of the profession. It is worth noting that the visual range of a postage stamp is estimated at 9,200,000 people. Its circulation as proof of payment for correspondence is limited by ECT, but in the field of philatelic commerce and collecting it has unlimited circulation worldwide, for years, decades and centuries.

As a public company and agent of the federal government’s public policies, ECT has fulfilled its mission to honor and publicize the nursing profession through the images and captions printed on postal documents. The iconological reading of the representations of nursing in Brazilian philately shows the value and public recognition, by the Brazilian State, through the Brazilian Post Office, of the social importance of the profession and nursing professionals in the health care of the Brazilian people.

It is hoped that other postal documents representing nursing will be issued, to remedy the absences felt and increase the visibility of the profession. However, the representation should be made up of real images and professional symbols, without random elements of nursing symbolism or the fragmentation of institutional and nursing professional associations logos. Anyone can send their proposal for a theme to the Brazilian Post Office, simply by filling in the form on the company’s website and attaching information about the suggested theme and its justification, as well as ordering a personalized postage stamp, negotiating with the Post Office Manager the quantity to be issued and the amount to be paid.
