# Supplementary material for: Panofskyan iconological reading of images representing nursing in Brazilian philately
Source: Rev Esc Enferm USP. 2025 Feb 7;58:e20240284. doi: 10.1590/1980-220X-REEUSP-2024-0284en (PMC11804659; doi:10.1590/1980-220X-REEUSP-2024-0284en)
Supplement: Translation

# Translation: Leitura iconológica panofskyana das imagens de representação da enfermagem na filatelia brasileira*

**DOI:** 10.1590/1980-220X-REEUSP-2024-0284pt

**Authors:** Anesilda Alves de Almeida Ribeiro, Genival Fernandes de Freitas

**Affiliations:** 1 Universidade de São Paulo, Escola de Enfermagem, Departamento de Orientação Profissional, São Paulo, SP, Brasil.

**Keywords:** Enfermagem, história da enfermagem, ciência nas artes, desenho, filatelia

## Abstract

**Objetivo:**

Analisar as imagens dos documentos postais brasileiros com representação da enfermagem.

**Método:**

Pesquisa histórica com recorte temporal entre 1843 e 2022. Os dados foram coletados pela técnica de observação visual, do acervo iconográfico do Museu dos Correios e catálogos filatélicos. A análise dos desenhos postais seguiu o Método Iconológico de Erwin Panofsky.

**Resultados:**

Foram identificados 62 documentos postais alusivos à enfermagem. A arte gráfica foi composta por elementos da simbologia da profissão, personalidades, organizações sociais, categorias profissionais, ações de cuidado, instituições de saúde, entidades de classe, eventos científicos e escolas de enfermagem. Observou-se ausência de temas acadêmicos, enfermeiras pioneiras e revistas de enfermagem.

**Conclusão:**

Desde 1934, a enfermagem é representada na filatelia brasileira, independentemente do contexto político. As representações evidenciam o reconhecimento público, pelo Estado brasileiro, da importância social da profissão e dos profissionais de enfermagem no cuidado à saúde humana. O estudo inovou a pesquisa em enfermagem no Brasil ao usar o selo postal como fonte de dados e a metodologia panofskyana na leitura de imagens da profissão.

## INTRODUÇÃO

A imagem é uma representação e um veículo de comunicação, com função, história e significado. Dentro da história da arte, distintos temas podem ser representados por imagens e construídos por variadas técnicas. O tema da enfermagem foi explorado por inúmeros artistas visuais, sendo a imagem da profissão expressa de forma realista e estilizada em obras de arte do tipo figurativa, como pintura, escultura, fotografia, gravura, aquarela, ilustração e desenhos, especialmente os impressos em selos postais.

O selo postal foi criado em 1840, na Inglaterra, para servir como comprovante de pagamento no envio de correspondência. O Brasil foi o segundo país do mundo a emitir o selo postal, fato ocorrido em 1º de agosto de 1843. O selo postal brasileiro é impresso na Casa da Moeda e emitido pela Empresa Brasileira de Correios e Telégrafos (ECT), vinculada ao Ministério das Comunicações. A ECT emite outros documentos postais, como carimbos comemorativos, bilhetes postais, envelopes de primeiro dia (EPD) e folhas comemorativas. Compete à Comissão Filatélica Nacional, composta por representantes do governo federal e das instituições públicas supracitadas, escolher os temas que comporão o Programa de Selos Postais de cada ano. As imagens são criadas por artistas plásticos, sendo compostas por elementos textuais e gráficos, e preenchimento em cores variando do monocromático ao multicolorido^(1)^.

Até meados do século XX, os selos postais serviram exclusivamente como objetos de colecionismo e produtos de comercialização filatélica. Desde 1997, foram tomados como fonte de dados de pesquisa. Revisão integrativa de literatura identificou o uso de selo postal por pesquisadores brasileiros de várias ciências, mas nenhum estudo na área da saúde. A produção nacional, constituída por artigos, trabalhos de conclusão de curso, monografias, dissertações e teses, destaca o valor do selo postal na pesquisa acadêmica, por conter registros e divulgar ideias e acontecimentos sociais, científicos e históricos relevantes^(2)^.

A história postal universal registrou a imagem de várias profissões, inclusive da enfermagem. Os desenhos filatélicos da enfermagem têm sido utilizados, desde 1941, como objeto de investigação por pesquisadores internacionais. O primeiro artigo científico sobre essa temática foi publicado no *American Journal of Nursing*, dos Estados Unidos da América (EUA), e contou com um selo postal brasileiro^(3)^. Estudo mostra que o marco inicial de representação de uma Enfermeira real e identificada nominalmente na filatelia latino-americana foi o ano de 1957^(4)^. Artigo biográfico de enfermeira laica americana foi ilustrado com selo postal com seu retrato^(5)^. Enfermeiras religiosas, militares e fundadoras de escolas de enfermagem foram homenageadas em selos postais^(6)^. Estudo da Costa Rica identificou a trajetória da enfermagem local em selos postais^(7)^. A filatelia venezuelana divulgou a identidade da enfermagem de instituições religiosas, humanitárias e hospitalares^(8)^. Estudos da filatelia argentina apontam formas distintas de representação da enfermagem^(9)^, e um quantitativo minoritário de Enfermeiras e Médicas em comparação a de Médicos^(10)^.

Da Europa, destacam-se os estudos filatélicos da Enfermeira e filatelista Dra. Maria Teresa Miralles Sangro, publicados no formato de artigo, livro e tese de doutorado. Analisando sua coleção de documentos postais, a autora citada identificou fatos históricos, personalidades, datas comemorativas, uniformes, símbolos e cuidados de enfermagem em selos postais de mais de 130 países, incluindo o Brasil^(11–14)^.

Há mais de 80 anos o selo postal brasileiro serve de objeto de estudo científico na pesquisa médica e de enfermagem internacional. Apesar disso, ainda não se tinha nenhum estudo da enfermagem brasileira sobre a imagem da profissão nas emissões postais da ECT. Diante do exposto, desenvolveu-se uma pesquisa com a questão norteadora: quais as representações da enfermagem na filatelia brasileira e seu significado? Objetivo: analisar as imagens dos documentos postais brasileiros com representação da enfermagem^(15)^.

## MÉTODO

### Tipo de Estudo

Pesquisa de abordagem histórica, no campo da História das Ciências da Saúde e domínio da História da Enfermagem.

### Local, População, Critérios de Seleção e Definição da Amostra

A pesquisa foi realizada em Itajubá/MG e na internet. A população compreendeu a totalidade de documentos postais emitidos pela ECT. O critério de seleção incluiu os documentos postais brasileiros, emitidos no recorte temporal de 1843 até 2022, e com imagem composta por elementos alusivos à profissão de enfermagem. Na definição da amostra foram excluídos os documentos postais com imagem repetida ou discreta variação no desenho ou legenda, os relacionados à doença e os sem a representação da enfermagem.

### Coleta de Dados

Os dados foram coletados pela primeira autora, no período de janeiro a dezembro de 2022, pela Técnica de Observação Visual, na sua coleção particular de selos postais e de material impresso – Catálogo Zioni-Soares^(16)^ e Catálogo de Selos do Brasil^(1)^, e junto ao acervo digital do Museu dos Correios.

### Análise e Tratamento dos Dados

A análise foi guiada pelo referencial teórico-metodológico do historiador de arte alemão Erwin Panofsky (1892–1968), criado para o estudo do significado nas Artes Visuais, que fazem alusão à realidade cotidiana. Panofsky afirma que a obra de arte é uma representação que traz a visão de mundo do seu criador e o contexto histórico de sua criação. O referencial panofskyano utiliza dois conceitos: Iconografia, relativo à descrição e classificação dos elementos constitutivos da composição artística; e Iconologia, que trata da interpretação da mensagem intrínseca, ou seja, do significado total da obra de arte^(17)^.

O tratamento dos dados seguiu o Método Iconológico criado por Erwin Panofsky^(17)^, caracterizado pela aplicação sequencial de três categorias: 1) Descrição Pré-Iconográfica, 2) Análise Iconográfica; e 3) Interpretação Iconológica. O estudo iniciou pela descrição dos elementos constitutivos da imagem e suas relações; seguiu pela análise do tema da representação, identificando o criador e o contexto histórico da arte gráfica, a biografia de personalidades, o histórico da organização ou instituição representada; e finalizou com a interpretação do significado das representações visuais da enfermagem na filatelia brasileira.

### Aspectos Éticos

A pesquisa não envolveu seres humanos. O projeto foi aprovado pelo Comitê de Ética em Pesquisa da Escola de Enfermagem da Universidade de São Paulo, Parecer nº 4.420.440, em consonância com a Resolução nº 510/2016, do Conselho Nacional de Saúde, relativa ao uso de imagem. A Gerência de Filatelia dos Correios autorizou o uso e reprodução das imagens para fins de pesquisa e publicação^(15)^.

## RESULTADOS

Foi metodicamente observado um quantitativo de 17.670 documentos postais (11.144 carimbos, 4.930 selos, 1.302 EPD, 249 bilhetes e 45 folhas). A aplicação dos critérios de inclusão selecionou 156 imagens e dos critérios de exclusão resultou em uma amostra com 62 documentos postais, sendo: 31 carimbos comemorativos, 22 selos postais, cinco EPD, duas folhas comemorativas e dois bilhetes postais^(15)^.

As imagens elencadas para o estudo foram classificadas por similaridade, dando origem a quatro grupos temáticos: 1) personalidades e organizações sociais de enfermagem (15 imagens), 2) cursos e escolas de enfermagem (oito imagens), 3) eventos científicos e entidades de classe da enfermagem (26 imagens), e 4) enfermagem em ações de cuidado em instituições de saúde (13 imagens).

Pela limitação de espaço, são apresentadas as imagens mais simbólicas de cada grupo temático, agrupadas em quatro figuras, distribuídas e analisadas conforme a ordem cronológica de emissão pela ECT. As demais imagens da amostra e outras informações podem ser acessadas no documento de tese^(15)^.

### Personalidades e Organizações Sociais de Enfermagem

A filatelia brasileira representou cinco personalidades da enfermagem, sendo três brasileiras (Padre José de Anchieta, Ana Justina Ferreira Neri e Padre Bento Dias Pacheco) e duas estrangeiras (São Vicente de Paulo e Florence Nightingale), e duas organizações sociais de enfermagem: Cruz Vermelha e Filhas da Caridade (Figura 1).

**Figura 1:** Imagens postais de personalidades e organizações sociais de enfermagem.

Fonte: Correios.

Padre José de Anchieta nasceu em 1534, na Espanha, estudou em Portugal na Companhia de Jesus, veio para o Brasil em 1553, onde trabalhou como catequizador, professor, escritor e Enfermeiro, e faleceu em 1597. Anchieta é reconhecido pela literatura de história da enfermagem como o primeiro Enfermeiro do Brasil^(18)^. O IV centenário de seu nascimento, celebrado em 1934, contou com emissão de selo postal. A imagem, criada por Leopoldo Alves Campos, é composta pela representação desse santo católico usando batina, carregando a cruz processional e prestando cuidado espiritual a indígena do litoral brasileiro. A ECT emitiu outros selos postais de Anchieta, todos enaltecendo seu papel como sacerdote. Estudo argentino também identificou que personagens históricos da saúde nem sempre são retratados na filatelia pelo exercício da profissão, mas por outras atividades, dentre as quais as religiosas^(10)^.

A organização social Cruz Vermelha foi criada em 1863, na Suíça, hoje presente em 190 países, sendo a unidade brasileira fundada em 1908. O evento 3ª Conferência Pan-Americana da Cruz Vermelha, realizado em 1935, no Rio de Janeiro/RJ, foi tema de selo postal. A imagem, criada por Marino Ferreira Pinheiro, é composta por cenário de guerra, com Enfermeira estilizada, usando uniforme longo com avental e véu, amparando nos braços um soldado ferido. A arte gráfica é considerada belíssima, incomum e símbolo dos ideais da enfermagem, de acolher e cuidar do próximo, sendo recorrente na literatura científica internacional, especialmente na que apresenta selos postais alusivos à enfermagem^(3,11,12)^.

As Filhas da Caridade, organização religiosa católica feminina, criada em 1633, na França, por São Vicente de Paulo e Luiza de Marillac, se espalhou por cinco continentes, chegando ao Brasil em 10 de fevereiro de 1849. O 1º Centenário da chegada dessas religiosas ao país foi celebrado com emissão de folha comemorativa, emitida em 1950. A imagem, criada por Bernardino da Silva Lancetta, é composta pela representação de Irmãs de Caridade com traje longo e véu tipo corneta. No contexto da emissão postal, enfermeiras vicentinas brasileiras se dedicavam à educação em saúde, ao cuidado domiciliar de pobres e doentes, chefiavam instituições de saúde e dirigiam escolas de enfermagem do tipo confessional católica^(18)^.

Ana Neri nasceu na Bahia, em 1814, e faleceu no Rio de Janeiro em 1880. Ela prestou serviços de enfermagem junto ao corpo de saúde do Exército brasileiro durante a Guerra do Paraguai (1864–1870), conflito do qual participaram alguns de seus irmãos e filhos^(18)^. Esse vínculo com as Forças Armadas lhe rendeu uma homenagem em 1967, quando a ECT emitiu selo postal e um EPD com a legenda: Ana Justina Ferreira Neri – 1ª Enfermeira do Brasil. A imagem do selo postal, criada por Waldomiro Puntar, é composta pelo retrato dessa ilustre personalidade da enfermagem brasileira. O contexto da emissão foi a Ditadura Militar (1964–1985) e a homenagem às Mulheres Famosas do Brasil. Apesar de emitido no dia 12 de maio, o edital de lançamento não menciona a celebração do Dia da Enfermeira, mas apresenta a biografia dessa celebridade, única Enfermeira brasileira identificada nominal e visualmente em selo postal, até o momento.

O IV Centenário de nascimento de São Vicente de Paulo (1581–1660), celebrado em 1982, contou com emissão de selo postal. A imagem, criada por Jorge Eduardo Alves de Souza, é composta pela reprodução de uma pintura do século XVII, de Simon François, destacando a figura desse religioso católico cuidando de um cidadão francês doente e desabrigado. O edital de lançamento traz uma síntese biográfica desse santo da Igreja Católica, membro da Ordem de São Francisco de Assis, que dedicou a vida à caridade, criou congregações masculina e feminina como os Padres Lazaristas e as Filhas da Caridade, e teve propagado o seu carisma vicentino de cuidado caridoso aos pobres em 150 países, inclusive no Brasil^(18)^.

A Semana de Combate à Hanseníase, de 1984, contou com emissão de selo postal com a efígie do Padre Bento Dias Pacheco (1819–1911). A imagem, criada por Martha Poppe, é composta pelo perfil desse religioso católico usando batina e o vitral de uma igreja. A literatura de enfermagem desconhece a obra dessa personalidade histórica, mas o Estado brasileiro, através dos Correios, lhe atribuiu o título de Enfermeiro, pelos cuidados prestados a hansenianos de Itu/SP, conforme descrito no edital de lançamento.

Florence Nightingale nasceu em 1820 e faleceu em 1910. Essa enfermeira inglesa organizou o serviço de enfermagem britânico na Guerra da Criméia (1853–1856), fundou a enfermagem moderna e a primeira escola de enfermagem mundial com base no rigor técnico científico. Ela divulgou a profissão e o cuidado de enfermagem por meio de cartas, notas, relatórios e livros^(9,18)^. O bicentenário de seu nascimento, celebrado em âmbito mundial em 2020, foi divulgado no Brasil através de emissão de selo postal. A imagem é composta por seu retrato e assinatura. A arte gráfica foi cedida pelo Conselho Regional de Enfermagem da Bahia (Coren-BA), se trata de um selo postal personalizado, emitido sob encomenda, com circulação normal, em âmbito nacional, nas correspondências da autarquia e venda a filatelistas e colecionadores.

### Cursos e Escolas de Enfermagem

A ECT emitiu documentos postais de sete Instituições de Ensino Superior (IES), pública ou privada, federal ou estadual (Figura 2).

**Figura 2:** Imagens postais de cursos e escolas de enfermagem.

Fonte: Correios.

A Escola de Enfermagem Alfredo Pinto (EEAP) foi criada em 1890, no modelo francês, com direção e ensino por médicos. Em 1943, essa IES pública federal do Rio de Janeiro, pioneira na formação de Enfermeiros e Enfermeiras, admitiu Enfermeiras na direção e corpo docente^(18)^. Em setembro de 1965, a ECT emitiu dois carimbos comemorativos da EEAP. O carimbo no formato retangular traz legenda informando a carência de Enfermeiras no país e a abertura de matrículas pela Escola. O carimbo circular foi emitido em celebração dos 75 anos da Escola, sendo a imagem composta pelo desenho da lamparina, alusiva à lâmpada símbolo da Enfermagem. Essas emissões postais inauguraram o marketing das escolas de enfermagem através da filatelia brasileira.

A Escola de Enfermagem da Universidade de São Paulo (EEUSP), criada em 1942, teve por primeira diretora a Enfermeira Edith de Magalhães Fraenkel (1889–1968), diplomada nos EUA^(19)^. Desde os primórdios, essa IES pública estadual é um centro de excelência na formação profissional e na pesquisa em enfermagem. Os 50 anos da EEUSP, celebrados em 1992, contou com emissão de carimbo comemorativo, com imagem composta pela logomarca de celebração, constituída pelo Logotipo USP, sem a presença de símbolos da enfermagem ou a identidade visual da Escola – Logotipo e Brasão. No contexto do seu Jubileu de Ouro, a instituição celebrou os 25 anos da Revista da Escola de Enfermagem da USP (REEUSP) e inaugurou o Centro Histórico-Cultural da Enfermagem Ibero-Americana (CHCEIA), responsáveis pela preservação do acervo da memória histórica da Escola.

A Escola de Enfermagem Anna Nery (EEAN) foi criada em 1923, com o objetivo de diplomar Enfermeiras e difundir o modelo anglo-americano de ensino da enfermagem moderna a nível nacional, sendo este caracterizado por ter Enfermeira na direção escolar e nas salas de aula, e o ensino em regime de internato. Até 1931, a instituição foi dirigida por enfermeiras americanas, após assumiram a direção Enfermeiras brasileiras. Em 1937, a Escola foi incorporada à Universidade Federal do Rio de Janeiro (UFRJ). Em 1971, passou a oferecer curso de enfermagem misto^(18)^. Os 70 anos da EEAN, celebrados em 1993, contou com a emissão de carimbo comemorativo. A imagem é composta pela reprodução da fachada do Pavilhão de Aulas da instituição, cenário de grandes lutas e conquistas da enfermagem brasileira.

A Escola de Enfermagem da Universidade Federal Fluminense (EEUFF), criada em 1944, teve por primeira diretora a Enfermeira Aurora de Afonso Costa (1903–1999), em cuja gestão ocorreu a federalização e implantação dos cursos de especialização. Em 1994, na celebração do seu Jubileu de Ouro, essa IES foi renomeada para Escola de Enfermagem Aurora de Afonso Costa (EEAAC) e a ECT emitiu um carimbo comemorativo alusivo aos seus 50 anos de criação. A imagem é composta pela figura geométrica de um triângulo e a lâmpada da enfermagem em seu interior.

A Escola de Enfermeiras Rachel Haddock Lobo (EERHL) foi criada em 1948, recebendo o nome em homenagem a Rachel Haddock Lobo (1891–1933), enfermeira brasileira pioneira, diplomada na França e com complementação curricular na EEAN. Em 1968, essa IES foi renomeada para Faculdade de Enfermagem da Universidade Estadual do Rio de Janeiro (UERJ). A comemoração dos 50 anos da Escola, em 1998, contou com emissão de bilhete postal. A imagem, criada por Carlota Rios, é composta pela fachada das três sedes da Escola. A legenda no verso da emissão informa que essa IES tem contribuído, desde a sua criação, para a saúde, o desenvolvimento e a cidadania no Rio de Janeiro.

A Escola de Enfermagem de Ribeirão Preto da USP (EERP-USP), criada em 1953, como anexo da Faculdade de Medicina de Ribeirão Preto, teve por primeira diretora a Enfermeira Glete de Alcântara (1910–1974), diplomada em Toronto, Canadá. Por força estatutária, em alguns momentos históricos essa IES foi dirigida por Médicos e Engenheiro. Desde 1986, está sob a direção de Enfermeiros. Os 50 anos da EERP-USP, comemorados em 2003, contou com emissão de carimbo comemorativo. A imagem, criada por Solange Aparecida da Silva e Rogério Cândido Ribeiro, é constituída pela logomarca de celebração do cinquentenário da Escola, composta pelo desenho de um arco com 13 estrelas de tamanhos diferentes, sem a simbologia da enfermagem ou o belíssimo Logotipo institucional. No contexto do Jubileu de Ouro foram comemorados os 10 anos da Revista Latino-Americana de Enfermagem (RLAE), editada pela Escola.

Os 25 anos do Curso de Enfermagem da Universidade do Vale do Itajaí (Univali), comemorado em 2005, contou com emissão de carimbo comemorativo. A imagem, criada por Nelenize Heyse Moreira, é composta pela logomarca da Univali, cinco estrelas e a lâmpada no modelo criado para compor a logomarca da Associação Brasileira de Enfermagem (ABEn), sendo esta usada indevidamente e de forma fragmentada, pois a imagem foi criada sob encomenda da ABEn para servir como sua marca registrada^(15)^. A Univali é uma IES privada, com sede em Itajaí/SC, composta pela Escola de Ciências da Saúde, da qual o Curso de Enfermagem foi o pioneiro. No contexto do Jubileu de Prata do curso, a ECT emitiu também um selo postal personalizado que foi aplicado nos diplomas dos formandos daquele ano.

### Eventos Científicos e Entidades de Classe da Enfermagem

O serviço postal brasileiro emitiu documentos postais com imagens alusivas a três eventos científicos e três entidades de classe da enfermagem brasileira (Figura 3).

**Figura 3:** Imagens postais de eventos científicos e entidades de classe da enfermagem.

Fonte: Correios.

O 10º Congresso Internacional de Enfermagem, promovido pelo Conselho Internacional de Enfermeiras, realizado em 1953, no Rio de Janeiro, contou com emissão de EPD, composto por selo postal e carimbo comemorativo. A imagem do EPD, criada por Waldomiro Puntar, é composta por uma Enfermeira moderna anônima usando uniforme branco e a touca da enfermeira, ladeada por folhas na cor verde. O selo, na cor verde, traz o desenho da Autoestrada Rio Petrópolis, Serra do Mar e a lâmpada grega. A legenda do carimbo comemorativo informa a data do evento. Essas emissões postais marcam o primeiro registro na história postal brasileira de duas simbologias da enfermagem – lâmpada e a cor verde – e de dois símbolos socialmente relacionados à profissão – uniforme branco e a Touca da Enfermeira.

A Semana da Enfermagem é o primeiro evento científico da enfermagem brasileira. Foi instituída em 1940, por Laís Netto dos Reys, diretora da EEAN, sendo celebrada de 12 a 20 de maio^(18)^. O evento faz parte do calendário comemorativo de instituições de ensino e saúde, e sua programação conta com homenagens a profissionais ilustres, atividades culturais, científicas e de divulgação da profissão^(18)^. A Semana da Enfermagem da ABEn-Seção Guanabara (ABEn-GB), de 1967, contou com emissão de carimbo comemorativo, no formato quadrado, com imagem composta pela lâmpada grega com a chama acesa e raios.

A ABEn, criada em 1926, é a primeira entidade de classe da enfermagem brasileira, responsável pela promoção cultural, científica e política da profissão. Sua primeira presidente foi Edith de Magalhães Fraenkel^(18)^. Os 50 anos da ABEn, celebrados em 1976, contou com emissão de selo postal. A imagem, criada por Raul Rangel, é composta pelo perfil do Enfermeiro e a logomarca da ABEn, constituída pela lâmpada grega com a chama acesa e quatro estrelas, alusivas à Constelação Cruzeiro do Sul.

O Congresso Brasileiro dos Conselhos de Enfermagem (CBCENF) é um evento do Conselho Federal de Enfermagem (Cofen) e do Coren ou Sistema Cofen/Corens^(20)^. A primeira edição, realizada em 1998, contou com emissão de bilhete postal. A imagem é composta pela representação dos mapas e bandeiras do Brasil, Panamá, Cuba, EUA, Dinamarca e Suíça, países de origem das participantes. Setas indicam a cidade onde foi realizado: Natal/RN. A entidade promotora é indicada pelo Brasão do Cofen, criado por Ori Ramos, sendo este constituído pela lâmpada da enfermagem, bastão serpentário da saúde e cinco estrelas, alusivas às cinco regiões do país onde estão instaladas as unidades dos Corens.

Os 25 anos do Sindicato dos Enfermeiros do Estado de São Paulo (SEESP), celebrado em 2010, contou com emissão de carimbo comemorativo. A imagem, criada pela RS Press Comunicações, é composta pela cruz grega, formada por dois braços do mesmo tamanho que se cruzam no centro, num ângulo reto.

O Cofen, criado em 1973, é responsável pela fiscalização do exercício profissional da enfermagem brasileira. A primeira presidente foi a Enfermeira Maria Rosa Sousa Pinheiro (1908–2002), diplomada em Toronto, Canadá^(20)^. Os 40 anos do Cofen, celebrados em 2014, contou com emissão de selo postal. A imagem, criada por Sandy de Assis Andrade, é composta pela logomarca de celebração, formada pelo numeral 40 desenhado de forma estilizada e o Brasão do Cofen. A imagem foi preenchida na cor azul, embora o Cofen tenha estabelecido a cor verde como símbolo da profissão de enfermagem.

### Enfermagem em Ações de Cuidado em Instituições de Saúde

A ECT emitiu documentos postais sobre o cuidado de enfermagem realizado na Atenção Primária à Saúde (APS) e secundário e terciário realizado em instituições hospitalares, privada e pública (Figura 4).

**Figura 4:** Imagens postais de enfermagem em ações de cuidado em instituições de saúde.

Fonte: Correios.

As imagens dos dois selos postais da vacinação infantil, criadas em 1983, por Rachel Braga, são compostas pela representação da mão da Técnica de Enfermagem. A identificação dessa categoria profissional baseou no disposto na Lei do Exercício Profissional, sendo a administração de medicação de competência da Técnica de Enfermagem^(20)^. As imagens a mostram aplicando a vacina contra o sarampo em uma menina, em via intramuscular, por meio de seringa de vidro; e administrando a vacina oral contra a poliomielite, por meio da gota do imunizante pingada diretamente na boca de um menino. Esses selos postais foram usados pelo Ministério da Saúde (MS), em parceria com o Ministério das Comunicações e ECT, para divulgar as campanhas de vacinação, visando à sensibilização das famílias e aumento da cobertura vacinal da população. Através da adesão ao Programa Nacional de Imunizações, a enfermagem da APS cumpriria a meta estabelecida no Calendário de Vacinação e o MS controlaria as doenças imunopreveníveis^(18)^.

A Santa Casa de Misericórdia de Juiz de Fora, criada em 1854, é vinculada à Irmandade de Nosso Senhor dos Passos. No início, o serviço de enfermagem da instituição era realizado por Enfermeiras religiosas católicas, da Congregação de Santa Catarina, originárias da Alemanha. Depois, o gerenciamento de enfermagem passou para Enfermeiras laicas^(15)^. Os 140 anos da instituição, celebrados em 1994, contou com emissão de carimbo comemorativo. A imagem é constituída pela logomarca da instituição, composta pela cruz grega e uma Enfermeira religiosa com hábito branco, véu, crucifixo e uma criança nos braços.

A Maternidade Carmela Dutra foi criada em 1955, em Florianópolis/SC. Essa instituição pública de saúde contou com o serviço de capelania de padres jesuítas, o gerenciamento das Irmãs da Divina Providência e parteiras na sala de parto. Na década de 1960, foi contratada a primeira Enfermeira religiosa diplomada. Em 1972, ingressou no serviço de enfermagem uma Enfermeira obstétrica laica^(21)^. Os 40 anos da maternidade, celebrados em 1995, contou com emissão de carimbo comemorativo. A imagem é composta pela parteira ou obstetriz, ladeada por um Médico obstetra e uma mãe pegando o filho nos braços.

A Sociedade Beneficente de Senhoras do Hospital Sírio-Libanês foi criada em 1921, por Adma Jafet e senhoras da comunidade árabe da capital paulista, com a finalidade de servir como mantenedora do Hospital, uma instituição privada, de excelência, constituída por Técnicos de Enfermagem e Enfermeiros qualificados que oferecem cuidados de enfermagem com qualidade e segurança aos pacientes^(15)^. Os 85 anos da Sociedade, celebrados em 2006, contou com emissão de carimbo comemorativo. A imagem, criada pela Agência Ogilvy Brasil de publicidade, traz a reprodução da primeira logomarca da instituição, composta pela cruz grega, raios e uma Enfermeira com uniforme branco de manga longa, avental e véu.

As imagens dos três selos postais de combate à COVID-19, criados em 2020, por Alan Magalhães, são compostas pela representação de profissionais de saúde da atualidade, não reais, usando conjunto de calça e camisa colorida e sapato fechado. Do selo postal acima e a esquerda destaca-se a imagem da Enfermeira socorrista, branca, usando o uniforme azul do Serviço de Atendimento Móvel de Urgência (SAMU), composto pelo macacão com estrela da vida e tarjas refletivas, boné e mochila na cor vermelha. Do selo postal da direita destacam-se a Auxiliar de Enfermagem, negra, com uniforme rosa, óculos, máscara e papeleta; o Técnico de Enfermagem, branco, com uniforme na cor verde escura e máscara; e o Enfermeiro, branco, com uniforme branco, usando os Equipamentos de Proteção Individual (EPI): máscara, Face Shield, óculos e luvas. O selo postal abaixo é composto pelo Enfermeiro intensivista, branco, cuidando de paciente na Unidade de Terapia Intensiva (UTI), ladeado por equipamentos e materiais de suporte avançado de vida.

As imagens dos dois selos postais das Vacinas foram criadas, em 2022, por Alan Magalhães. A da esquerda é composta pela representação da mão em luva da Técnica de Enfermagem aspirando imunizante do frasco de vacina por meio de seringa descartável e uma família de quatro pessoas. A imagem da direita é composta pela mão da Técnica de Enfermagem com uniforme verde, pistola com frasco de imunizante acoplado, administrando a vacina contra a varíola no braço direito da adolescente e ladeada por um casal idoso. A presença de pessoas de idades diferentes faz alusão ao direito de todos à vacina.

## DISCUSSÃO

Os resultados mostram que não houve nenhuma emissão postal com a temática enfermagem durante o governo de Pedro II (1840–1889) e primeira república (1889–1930). A partir da década de 1930 a enfermagem foi tema recorrente na filatelia brasileira, independentemente do contexto político. A primeira imagem, datada de 1934, traz a representação do Padre José de Anchieta. Em 1935, houve a representação de uma Enfermeira ficcional ou imaginária, em imagem estilizada alusiva à Cruz Vermelha.

Em 1967, uma Enfermeira real foi identificada visual e nominalmente em selo postal, trata-se de Ana Neri. Somente uma Enfermeira brasileira moderna, ou seja, diplomada, teve o nome registrado em documento postal, trata-se de Rachel Haddock Lobo, sendo sua identidade nominal relacionada a uma IES. Estudo em selos postais do continente americano identificou mulheres de várias profissões e ocupações, dentre as quais enfermeiras, médicas, rainhas, heroínas, escritoras, desportistas, engenheiras e governantes^(4)^.

Os termos Enfermeira e Enfermeiro aparecem em editais de lançamento de selos postais em 1935 e 1985, alusivos à Cruz Vermelha e ao Padre Bento Dias Pacheco, respectivamente.

Os elementos usados na composição das representações da enfermagem foram: Brasão do Cofen, logomarca da ABEn e os símbolos da profissão: lâmpada, seringa, cobra, cruz e a cor verde, sendo a simbologia profissional recorrente na filatelia mundial^(8,12–14)^. A Touca da Enfermeira e a cor azul constituíram-se símbolos secundários alusivos à enfermagem. A lâmpada, o uniforme profissional e a Touca da Enfermeira foram representados com variações de forma, modelo e cor. Foram utilizados elementos gráficos não relacionados à profissão, como arco, estrela e raio. A maior diversidade de elementos da composição foi observada nas imagens relacionadas à ABEn. O maior quantitativo de emissões foi sobre o Sistema Cofen/Conselhos Regionais, mas com repetição do Brasão institucional. As imagens mais realistas foram as com reprodução de fotografias de personalidades históricas e fachadas de sedes de IES.

A história postal brasileira registrou Enfermeiras laicas e voluntárias de guerra e Enfermeiros religiosos, de cor branca, que viveram no período em que a Enfermagem era prática e sem o domínio do saber científico^(1,18)^. As representações de Personalidades e Organizações Sociais de Enfermagem evidenciam o papel da enfermagem no contexto de paz e de guerra. A imagem de Florence Nightingale, São Vicente de Paulo, Filhas da Caridade e Cruz Vermelha, são recorrentes na filatelia internacional, devido ao reconhecimento de suas obras e contribuições, individual e coletiva, para a promoção da saúde humana^(4–14)^.

A filatelia brasileira emitiu documentos postais na celebração de aniversário de Cursos e Escolas de Enfermagem, compartilhando o orgulho nacional pela sua longevidade e papel na propagação da ciência, inovação e tecnologias da profissão. Essas representações revelam a importância das IES de enfermagem na formação das novas gerações de profissionais da assistência, gerência, docência e pesquisa. A filatelia mundial também rendeu homenagens a cursos e escolas de enfermagem pioneiras e centenárias^(12–14)^.

A ECT homenageou as Entidades de Classe da Enfermagem brasileira e divulgou seus eventos através da emissão de documentos postais contando a história do associativismo, fiscalização, sindicalismo e cientificidade profissional. Essas representações revelam a contribuição das entidades de classe na promoção científica, cultural, política, ética, legal e sindical da enfermagem. A filatelia mundial também emitiu selos postais divulgando associações, eventos internacionais e acontecimento históricos da enfermagem^(4–14,22)^.

Os Correios destacaram o trabalho da enfermagem realizado por profissionais, brancos e negros, das distintas categorias, em contextos e cenários diversos. As representações evidenciam o protagonismo da enfermagem e o valor do cuidado prestado na prevenção, promoção, recuperação e reabilitação da saúde, em todas as fases do ciclo vital e com o uso de tecnologia avançada. Ações de cuidado também são temas recorrentes na filatelia mundial, destacando a enfermagem da APS, rural, especializada e hospitalar^(3–14)^.

No contexto das emissões postais de 2020 e 2022, a enfermagem brasileira vivia as vicissitudes da pandemia de COVID-19, com taxa elevada de morbimortalidade de profissionais^(23)^ e ascensão do movimento antivacina. A enfermagem foi efetiva na linha de frente de enfrentamento e combate da doença, apesar das mudanças abruptas e perdas de vidas, recebendo por isso várias homenagens através de selos postais. Pelo ineditismo e gravidade da doença viral, o Estado brasileiro usou dessa mídia para homenagear os profissionais na linha de frente do enfrentamento à pandemia e propagar o valor da vacinação na prevenção de doenças, inclusive contra a COVID-19, reforçando a ideia de que vacina salva vidas.

Apesar de valorizados e fazer parte da filatelia mundial^(3–14,22)^, observou-se nos documentos postais brasileiros^(1,16)^ a falta de alguns temas ligados à enfermagem. Sentiu-se a ausência de representação do retrato e nome de profissionais reais do século XX e XXI; comemoração do centenário de nascimento de pioneiras e de teoristas de enfermagem; ritual do Juramento da Profissão, Recepção da Insígnia e Passagem da Lâmpada; cinquentenário de revistas de enfermagem e programas de pós-graduação; presidentes da ABEn e conselheiros do Cofen; Congresso Brasileiro de Enfermagem (CBEn); celebração da criação da Lei do Exercício Profissional e do Código de Ética da Enfermagem, Dia do Enfermeiro e Dia do Técnico e do Auxiliar de Enfermagem; salas de aula, eventos e jogos acadêmicos; ambientes de cuidado e práticas ritualísticas como administração de hemoderivados, coleta de material, curativos, bandagem, verificação de sinais vitais, exame físico; braçal da Cruz Vermelha; enfermeiros mártires e indígenas.

Como limitação do estudo destaca-se a não visualização de todos os documentos postais emitidos pelo serviço postal brasileiro, desde 1843, bem como dos editais de lançamento, devido a não preservação destes pela ECT e Museu dos Correios. E pela falta de informações sobre o histórico, identidade visual e logomarcas de celebração de algumas instituições de ensino, saúde e entidades de classe da enfermagem.

A pesquisa contribuiu para o entendimento do processo de escolha dos temas dos documentos postais e da importância da enfermagem ser representada neste tipo de mídia, sendo a profissão reconhecida pela filatelia brasileira como tema de relevância social e de interesse histórico. O Padre Bento Dias Pacheco foi apresentado como novidade da identidade profissional, mas sua biografia merece novas investigações. O estudo traz como inovação para a Pesquisa em História da Enfermagem no Brasil o uso dos documentos postais como fontes de dados e da metodologia panofskyana na leitura de imagens da profissão.

## CONCLUSÃO

A história postal brasileira celebrou de forma especial a profissão de enfermagem, divulgando suas principais marcas históricas através de imagens realistas, simbólicas e ficcionais. Apesar da aparição tardia e das ausências observadas, as representações deram visibilidade à identidade profissional, divulgaram a cientificidade, a simbologia e a história da enfermagem brasileira, homenagearam e imortalizaram: 1) Personalidades e Organizações Sociais de Enfermagem, 2) Cursos e Escolas de Enfermagem, 3) Eventos Científicos e Entidades de Classe da Enfermagem, 4) Enfermagem em Ações de Cuidado em Instituições de Saúde. As expressões artísticas sobre a enfermagem, materializadas nos documentos postais brasileiros, circularam o mundo e contribuíram para a criação de uma memória visual coletiva da profissão. Destaca-se que o alcance de visualização de um selo postal é estimado em 9.200.000 pessoas. Sua circulação como comprovante de pagamento de correspondência é limitada pela ECT, mas no ramo do comércio e colecionismo filatélico tem circulação ilimitada e em âmbito mundial, por anos, décadas e séculos.

Enquanto empresa pública e agente das políticas públicas do governo federal, a ECT cumpriu sua missão de honrar e divulgar a profissão de enfermagem, por meio de imagens e legendas impressas nos documentos postais. A leitura iconológica das representações da enfermagem na filatelia brasileira evidencia o valor e reconhecimento público, pelo Estado brasileiro, através dos Correios, da importância social da profissão e dos profissionais de enfermagem no cuidado à saúde do povo brasileiro.

Espera-se que sejam emitidos outros documentos postais com representação da enfermagem, para sanar as ausências sentidas e ampliar a visibilidade da profissão. Mas, que seja a representação composta por imagens reais e os símbolos profissionais, sem elementos aleatórios da simbologia da enfermagem e nem a fragmentação de logomarcas institucionais e de entidades de classe. Qualquer pessoa pode enviar sua proposta de tema aos Correios, bastando preencher formulário no *site* da empresa e anexar informações sobre o tema sugerido e sua justificativa e, também, encomendar selo postal personalizado, negociando com o Gestor da Agência de Correios a quantidade a ser emitida e o valor a ser pago.
